# Role of Interleukin-6 in Atherothrombosis and Myocardial Infarction

**DOI:** 10.1007/s11883-025-01387-8

**Published:** 2026-01-16

**Authors:** Camilla Huse, Ida Gregersen, Fredric A. Holme, Thor Ueland, Mona Skjelland, Anne Hege Aamodt, Tuva B. Dahl, Pål Aukrust, Bente Halvorsen

**Affiliations:** 1 Research Institute of Internal Medicine, Oslo University Hospital Oslo Norway, Oslo, Norway; 2 Institute of Clinical Medicine, Faculty of Medicine, University of Oslo Norway, Oslo , Norway; 3 Thrombosis Research Center (TREC), Division of Internal Medicine, University Hospital of North Norway, Tromsø, Norway; 4 Department of Neurology, Oslo University Hospital Rikshospitalet, Oslo, Norway; 5 Department of Neuromedicine and Movement Science, the Norwegian University of Science and Technology, Trondheim, Norway; 6 Institute of Population Health, Faculty of Health and Life Sciences, University of Liverpool, Liverpool, UK

**Keywords:** Interleukin 6, Inflammation, Myocardial infarction, Atherosclerosis, Atherothrombosis

## Abstract

**Purpose of the Review:**

We aimed to summarize the pathogenic role of IL-6 in atherothrombosis and myocardial infarction (MI), with focus on novel pathogenic mechanisms and current IL-6 targeted therapy in clinical cohorts.

**Recent Findings:**

IL-6 plays a major role in the pathogenesis of cardiovascular disease interacting with aging- and metabolic-driven inflammation, two conditions with overlapping molecular mechanisms. A novel pathogenic mechanism in cardiovascular disease (CVD) is the interaction between IL-6 and clonal hematopoiesis of indeterminate potential, CHIP, that involve the ten-eleven translocation-2 gene, a molecule with role in epigenetics. Recent studies showed reduced inflammation, a reduction in Lp (a) and inhibitory effects on platelets by anti-IL-6 (ziltivekimab) therapy in patients at risk for CVD. Anti-IL-6 receptor therapy (tocilizumab) showed reduced inflammation and improved myocardial function in MI patients, involving reduced degranulation in neutrophils and modulation of monocytes. Studies with clinical endpoints are still lacking and there also a need for developing therapy that selectively block the harmful IL-6 trans-signaling.

**Summary:**

IL-6 plays an important and many-faceted role in atherothrombosis including MI and ischemic stroke, and the IL-6 system represent a promising but still evolving therapeutic approach. A comprehensive understanding of the complexity of IL-6 signaling is needed to improve such treatment strategies.

## Introduction

The global incidence of cardiovascular disease (CVD) has risen significantly the last 30 years, by more than 70% increase between 1990 and 2019. Similarly, global CVD-related mortality increased by over 50% during the same period [1]. The highest rates are observed in regions with low- and middle sociodemographic indices. Of importance, age-adjusted rates of both incidence and mortality have had a steady decline in the same period [1]. However, the world’s aging population is growing, and CVD is and will be a major health problem world-wide in the years to come. Illustrating this, data from the American Heart Association show that heart disease and stroke were responsible for more deaths than cancers and chronic lower respiratory disease combined in the United States in 2022. Further, CVD accounted for 11% of the total US health expenditures in 2020–2021 [2]. These trends underscore the ongoing need for improved strategies in prevention, early detection and treatment of CVD.

## Treating Inflammation in Atherosclerotic disease – Interleukin-6 as a Target To Treat?

While the presence of inflammation in atherosclerotic lesions has been known for more than 100 years [3], in the 1980's Göran Hansson and colleagues demonstrated the presence of immune cells in human atherosclerotic plaque [4]. This was an important game changer in the view of inflammation and immune activation as a driving force in atherogenesis, operating in a bi-directional manner with lipid pathology [5]. During the last decades, extensive evidence has demonstrated a central role for inflammation in atherosclerosis with elevated levels of markers of inflammation and immune activation despite successful lipid lowering therapy [6]. This suggests a residual inflammatory risk that may be an important treatment target [7]. The CANTOS trial validated the clinical potential of anti-inflammatory treatment for atherosclerotic disease, by demonstrating that canakinumab, a human monoclonal antibody targeting interleukin (IL)−1β, can reduce major cardiovascular events [8]. Further, this reduction was directly associated with lowering of IL-6 levels [9], identifying IL-6 and related molecules as an additional potentially important target.

### IL-6 : a Role in Metaflammation and Inflammaging – Relevance for CVD

IL-6 is a cytokine that is produced by and has effects on both innate (e.g., monocytes/macrophage and dendritic cells) and adaptive (e.g., T cells and B cells) immune cells and exhibits both pro- and anti-inflammatory properties. In relation to vascular pathology, IL-6 is secreted by and modulate the function of several other cell types involved in atherothrombosis, including fibroblasts, vascular smooth muscle cells (VSMC), and endothelial cells, typically in response to IL-1 stimulation [10]. A broad range of additional stimuli can induce IL-6 release, such as tumor necrosis factor (TNF) and other inflammatory cytokines; toll-like receptor activation following activation by danger associated- or pathogen associated molecular patterns (DAMPs and PAMPs), prostaglandins and adipokines, and even psychological stress [11, 12]. In atherosclerotic heart disease, including myocardial infarction (MI), IL-6 is also produced by and have effects on cardiomyocytes [13, 14]. In ischemic stroke, both neurons, astrocytes and microglia cells can produce IL-6, indicating its broader involvement in neuroinflammation [15].

As a part of the body’s acute phase response, IL-6 signals the presence of inflammation and helps regulate immune responses [16]. IL-6 has a broad spectrum of biological functions, including stimulating the acute phase protein production in the liver, such as C-reactive protein (CRP), the promotion of B and T cell differentiation, regulation of fever and body temperature, maintenance of gut barrier integrity and support of hematopoiesis [11, 16, 17]. In the context of CVD, IL-6 contributes to angiogenesis, vascular remodeling and vascular calcification [16, 18]. In the low-grade inflammatory state, observed in metabolic disorders called metaflammation, IL-6 also has a metabolic role, including regulation of lipid metabolism and insulin resistance [19, 20]. Moreover, elevated IL-6 is associated with increased risk and severity of several age-related conditions, including atherosclerosis, diabetes, Alzheimer’s disease, and depression [21–23]. In relation to brain disorders, genetic variants that lead to increased IL-6 production have been linked to poorer cognitive outcomes and reduced longevity in age-related vascular disease [24]. Additionally, IL-6 is implicated in the pathogenesis of multiple autoimmune diseases such as rheumatoid arthritis, vasculitis and various auto-inflammatory disorders that all could accelerate atherogenesis [25] as well as certain types of cancer [26].

IL-6 has also been shown to influence hematopoiesis and the differentiation of hematopoietic progenitor cell. In aged bone marrow microenvironment, elevated IL-6 contribute to impaired erythropoiesis (red blood cell formation) and skews differentiation toward myeloid lineages, thereby increasing the overall inflammatory potential. Interestingly, inhibition of IL-6 signaling in aged mice has been found to restore erythroid progenitor function and enhance erythropoiesis, suggesting that IL-6 is a driver of age-associated hematopoietic dysfunction [27].

Taken together, these findings highlight IL-6 as a key contributor to **inflamm-aging**, the persistent chronic low-grade inflammation associated with aging and a wide spectrum of chronic diseases in older adults [12, 28, 29], closely linked to its role in metaflammation, two conditions with overlapping molecular mechanisms [30, 31].

Despite its well-documented pro-inflammatory effects, IL-6 also contributes to anti-inflammatory and tissue-reparative processes. It promotes expression of anti-inflammatory mediators such as IL-1 receptor antagonist (IL-1RA), IL-10 and soluble TNF receptors [17, 32]. Additionally, IL-6 seems to stimulate development of an anti-inflammatory and reparative phenotype in macrophages [28]. IL-6 is necessary for liver regeneration, and other reparative processes, indicating that complete inhibition of IL-6 signaling could impair recovery from injury or infection [24, 33] illustrating that both too little and too much IL-6 could be harmful.

## IL-6 Receptor Signaling – Different Effects of the Classical and Trans-signaling Route

IL-6 receptor (IL-6R) signaling is complex and regulated through different combinations of receptor units and different intracellular pathways, reflected in its wide range of biological effects. Membrane-bound IL-6R is expressed on a limited range of cells such as monocytes, lymphocytes, endothelial cells [10] and hepatocytes [34], whereas the co-receptor gp130 is ubiquitously expressed [35]. In addition to its-membrane bound form, IL-6R also exists in two soluble versions (sIL-6R), one generated by cleavage of the membrane-bound IL-6R, and the other is made through alternative splicing [36]. Gp130 also exists in a soluble form generated by alternative splicing [36], which specifically inhibits signaling mediated by the sIL-6R, but not the membrane bound IL-6R [37].

**Fig. 1:**
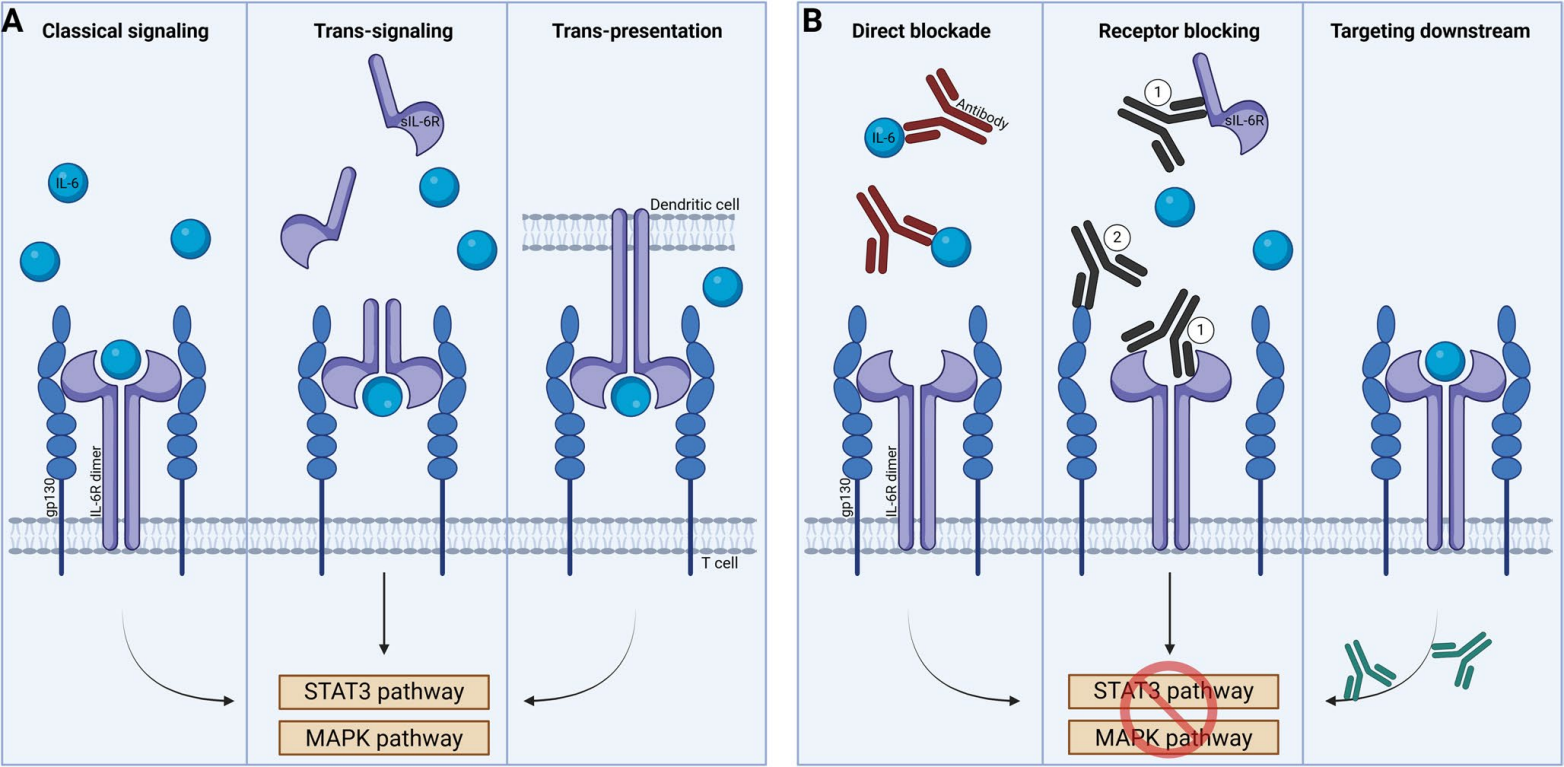
IL-6 signaling and routes of targeting. The left panel illustrates the three recognized modes of IL-6R signaling: classical signaling, trans-signaling, and trans-presentation. Classical IL-6 signaling involves the binding of IL-6 to the membrane-bound IL-6 receptor dimers of the membrane-bound IL-6R α-subunit and gp130. IL-6 trans-signaling involves complexes of IL-6 and the soluble form of IL-6 receptor (sIL-6R) signal via membrane-bound gp130, without involving membrane-bound IL-6R. In trans-presentation, a complex of IL-6 and membrane-bound IL-6R on dendritic cells is presented to gp130-expressing T cells. The right panel illustrates that targeting the IL-6 pathway can be divided into two interrelated categories: (**1**) antibodies blocking the IL-6R or IL-6, (**2**) antibodies blocking gp130. Modulation of these receptors will target downstream kinases or transcription factors in the JAK/STAT pathways. IL: interleukin, mAbs: monoclonal antibodies, JAK: Janus kinase, STAT: signal transducer and activator of transcription, MAPK: mitogen-activated protein kinase

There are three recognized modes of IL-6R signaling: classic signaling, trans-signaling, and trans-presentation (Fig. 1) [28, 35, 36]. Classical IL-6 signaling involves the binding of IL-6 to the membrane-bound IL-6R α-subunit and gp130 as the signal-transducing subunit. In IL-6 trans-signaling, complexes of IL-6 bound to sIL-6R signal via membrane-bound gp130, without involving membrane-bound IL-6R, resulting in the potential for induction of IL-6 signaling in a wide range of cell types [10, 35, 36]. Further, there has recently been identified a third mechanism of signaling, the trans-presentation [38]. In this signaling mode, the complex of IL-6 and membrane-bound IL-6R on dendritic cells is presented to gp130-expressing T cells; a process which seems to be required for priming of pathogenic T_H_17 cells, a subset of T cells implicated in autoimmunity and chronic inflammation [38].

Intracellular, the IL-6 responses is mediated through activation of Janus kinase (JAK) 1 and 2, and tyrosine kinase 2 (TYK2, JAK-family member) upon binding of IL-6 to IL-6R and the subsequent gp130 dimerization [36]. JAK auto-phosphorylation leads to activation of several intracellular signaling pathways, including MAP kinase and the signal transducer and activator of transcription 3 (STAT3) pathway. STAT3 activation mediates transcription of genes involved in recruitment, proliferation, differentiation, transformation, and survival [36]. As depicted in Fig. 1, targeting the IL-6 pathway can, based on the respective signaling pathways, be divided into three interrelated categories: (1) direct blockade of IL-6, (2) targeting a receptor such as IL-6R (including sIL-6R) or gp130 and (3) targeting downstream kinase or transcription factors in the JAK-STAT pathway. While both IL-6 classical and trans-signaling involve JAK/STAT pathways, the relative importance of the different transcriptional factor as well the involvement of Suppressor of Cytokine Signaling 3 (SOCS3), a major emulator of the intensity and duration of IL-6 responses [39], may differ somewhat between these two types of IL-6 signaling [40, 41].

During the past decade, IL-6 trans-signaling, targeting a wide range of cells that express gp130, has emerged as the predominant pathway by which IL-6 promotes disease pathogenesis. The classical signaling pathway also has the potential to promote anti-inflammatory responses. In trans-signaling, IL-6 seems to, at least in some degree to interact with IL-11, another member of the IL-6 family [42]. Importantly, whereas medication like tocilizumab (monoclonal antibody targeting IL-6R) and siltuximab (monoclonal antibody targeting IL-6R) inhibit IL-6 trans-signaling, they also inhibit IL-6 classical signaling. For, ziltivekimab (a monoclonal antibody targeting IL-6) we cannot find any precise data on targeted effects, but it is unlikely that this antibody will selectively block IL-6 trans-signaling. During the recent years, however, medications that selective block trans-signaling has been developed. Soluble gp130, has shown therapeutic potential in various preclinical models of disease and olamkicept, a sgp130Fc variant, had promising results in phase II clinical studies for inflammatory bowel disease [43, 44]. Data on selective blockade of IL-6 trans-signaling in CVD are scarce or lacking. However, in a model of reperfused MI in male rats, George MC et al., showed that sgp130Fc reduced infarct size and preserved cardiac function 28-days post-MI as compared with anti-IL-6 therapy [45]. They suggest that this difference was caused by selective blockade of trans-signaling and thereby more selective anti-inflammatory effects by the fusion-protein sgp130Fc.

## IL-6 in CVD – Mechanisms of Action and Possible Targets To Treat

**Fig. 2:**
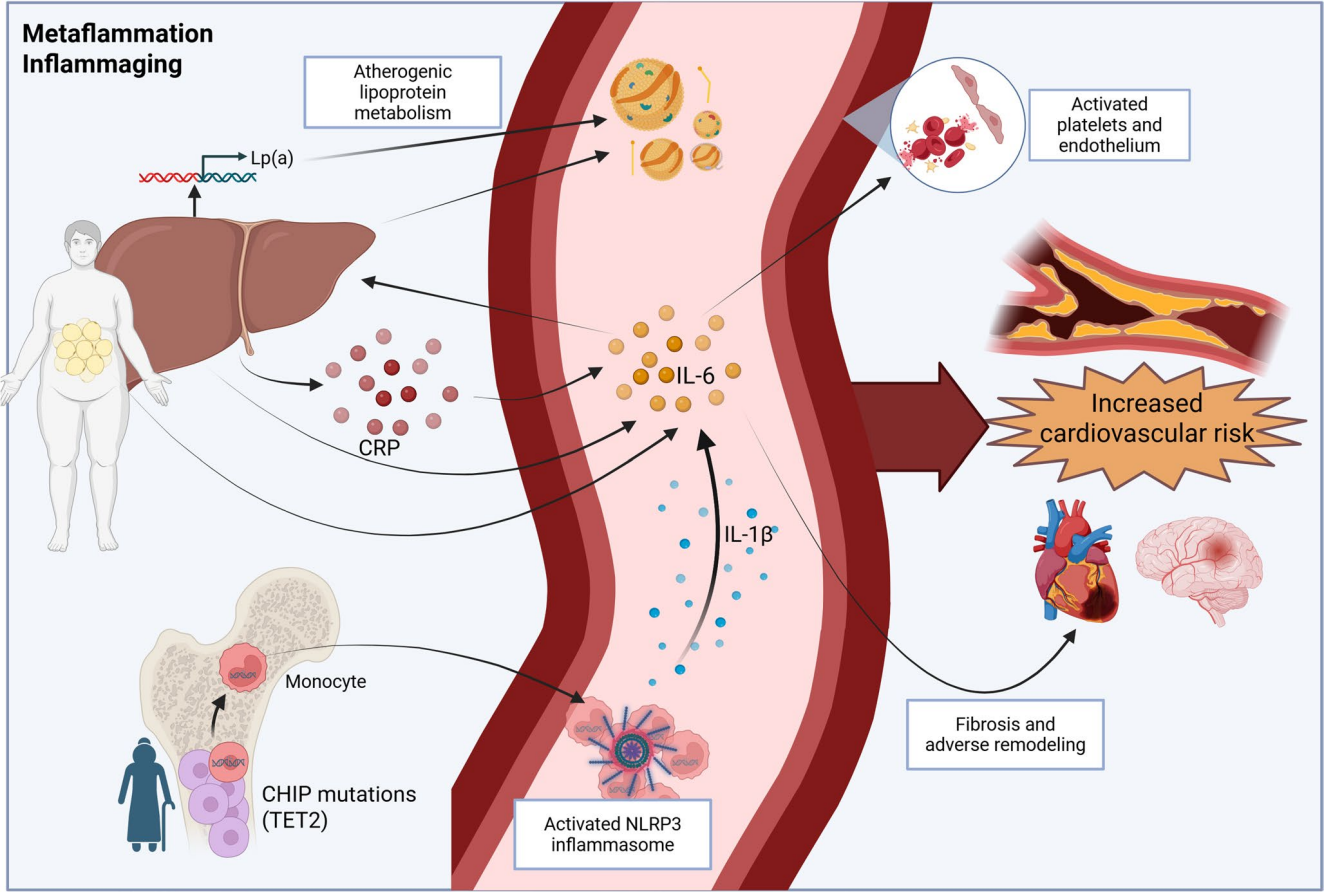
IL-6 in CVD – mechanisms of action. IL-6 has a broad range of interconnected effects of relevance to cardiovascular disease risk. IL-6 can promote an atherogeneic lipid profile (metaflammation) and is a central promotor of the increased cardiovascular risk along with aging (inflammaging). Its potent inflammatory effects are partly induced by NRLP3-inflammasome derived IL-1β. Clonal hematopoiesis of indeterminate potential (CHIP), especially those involving *ten–eleven translocation-2* (*TET2*) mutations, is in particular related to the harmful effects of IL-6 activation of the NLRP3 inflammasome and release of IL-1β result in production of IL-6. In turn, IL-6 stimulates production of CRP from the liver, further promoting inflammation. CRP can also influence IL-6 signaling by modulating the shedding of soluble IL-6R. IL-6 can further affect cardiovascular risk through lipid metabolism, promoting production of LP(**a**) and triglycerides (TG) from the liver, resulting in an atherogenic lipoprotein profile

Several reviews and metanalyses have demonstrated a strong association between elevated IL-6 levels and increased risk of adverse cardiovascular outcomes, including MI, heart failure, and ischemic stroke [46–50]. However, in the current review we will focus on novel mechanisms of IL-6 action (Fig. 2). and the potential for IL-6 targeted therapy in CVD.

### The Interaction between CHIP mutations, Inflammasome Activation and IL-6 in CVD

Clonal hematopoiesis of indeterminate potential (CHIP) refers to the age-related acquisition of mutations in hematopoietic stem cells, which results in the spread of mutated clones without overt hematological malignancies [39]. IL-6 plays an important role in the interaction between chronic inflammation and CHIP, especially those involving the *ten–eleven translocation-2* (*TET2*) gene, a particularly common CHIP mutation [51]. *TET2* is an important epigenetic regulator that controls DNA methylation and, thus, gene expression in response to the environment including aging [52]. Mutations in *TET2* gene impair its function, resulting in abnormal immune cell activity and an amplified inflammatory response, which includes increased IL-6 production [51]. Further, there is evidence that *TET2* expression can be influenced not only by mutation but also by epigenetic changes in myeloid cells, induced by metabolic dysregulation (e.g. obesity and diabetes) that can lead to prolonged activation of pro-inflammatory pathways [51, 53]. *TET2* mutations have been related to a significantly increased risk of CVD, regardless of traditional risk factors [54]. Interestingly, among carriers of large CHIP clones (DNMT3A or TET2), genetically reduced IL-6 signaling abrogated this risk. Thus, individuals who develop CHIP with simultaneous genetic deficiency of IL-6 signaling (by carrying IL6R p.Asp358Ala [instead of wild-type] as a proxy for inhibiting IL-6 signaling) had a greater reduction in CVD risk compared with those without CHIP with genetic IL-6 signaling deficiency [55]. Further, the CANTOS trial showed that the magnitude of benefit associated with targeted inflammasome inhibition (anti IL-1β treatment) was directly associated with the impact on IL-6 circulating levels and was especially effective in carriers of frequent CHIP mutant cells [9, 56].

In mice with experimental CHIP (tet2 deficiency) and hyperlipidemia, accelerated atherogenesis with heightened IL-1β/IL-6 signaling has been observed [57, 58]. In such models, inhibition of the NLRP3 inflammasome that activates and release IL-1β, ameliorates atherosclerosis in Tet2 deficient mice [58]. IL-6 is a known downstream target of IL-1β and is consistently increased in serum from patients with NLRP3 inflammasome-mediated conditions. The NLRP3-inflammasome and the IL-6 pathway seem to be linked by IL-1β-stimulating production of IL-6 [59]. Further downstream C-Reactive-Protein (CRP), produced in the liver as a response to IL-6, has been demonstrated to be a sustainable clinical marker of cardiovascular disease [59–61]. The NLRP3 inflammasome regulates the activation of caspase 1, which induces pyroptosis and activates the central proinflammatory cytokines IL-1β and IL-18 [61]. The mechanisms behind the regulation and activation of the NLRP3 inflammasome are not fully understood, but involve activation of TLR4-NFkB related pathways as a priming signal and for example potassium (K+) efflux, ATP and various crystals such as cholesterol crystals as activation signal 2 [62–64]. The CANTOS trial showed that canakinumab, a human monoclonal antibody blocking IL-1β, prevents recurrent cardiovascular events, indicating the NLRP3 inflammasome pathway to be of special importance. Indeed, the inflammasome signaling pathway was actively regulated in coronary thrombi and in circulating leukocytes from patients with ST-elevation myocardial infarction (STEMI), in association with myocardial damage measured by troponin T [59]. Moreover, we have demonstrated that cholesterol crystal-induced activation of NLRP3 inflammasomes are an important driver of both coronary and carotid atherosclerosis [65]. Importantly, NLRP3 inflammasome activation with enhanced IL-1β release will again enhance IL-6 production in a multitude of cells [66, 67].

Taken together, IL-6 and CHIP are related in that loss-of-function of the TET2 gene, is associated with increased IL-6 secretion, signaling and inflammation. These complex interactions also involve NLRP3 inflammasomes. Greater understanding of the processes that regulate TET2, or other CHIP, and its influence on IL-6 production by myeloid cells could offer valuable insights into the molecular mechanisms underlying chronic inflammation and CVD. Therapies that inhibit IL-6 signaling may be particularly effective in reducing cardiovascular risk among individuals with a high burden of CHIP, but prospective clinical trials are needed to confirm this hypothesis.

### The Role of IL-6 in Myocardial Remodeling after Myocardial Infarction

Whereas IL-6 seems to be harmful in relation to plaque destabilization and rupture, the phase of myocardial remodeling following MI is more complex. While inflammation has harmful effects in the initiation of MI, by contributing to plaque rupture, the degree of myocardial damage and the extent of ischemia-reperfusion (I/R) injury, inflammation also play an important role in the repair process following the initiating event [68–71]. The adult heart has little regenerative capacity, and healing of the infarcted myocardium is dependent on an orchestrated sequence of cellular events that lead to the formation of a collagen-based scar in which a balanced activation of pro-inflammatory and anti-inflammatory pathways plays an important role [72]. However, for the reparative process to be beneficial, the inflammatory response must be properly balanced with a timely repression of the inflammatory pathways for effective healing, followed by activation of infarct myofibroblasts that secrete matrix proteins in the infarcted area [71, 73]. A balanced inflammatory response is in itself critical for the activation of anti-inflammatory and pro-fibrotic pathways, including activation of transforming growth factor (TGF)β-related pathways, which will facilitate tissue repair and attenuate adverse post-MI remodeling [71, 74]. However, whereas an overwhelming and persistent post-infarct inflammation may lead to myocardial dilation and systolic dysfunction, an exaggerated pro-fibrogenic response may also be harmful by leading to development of diastolic dysfunction with a stiff myocardium [60, 75]. This dual role of inflammation following MI represents a therapeutic challenge.

The dual role of inflammation in MI development and myocardial remodeling is also clearly relevant for IL-6. Thus, whereas infusion of IL-6 in pre-clinical models have shown to promote myocardial hypertrophy, deficiency of its receptor induces severe cardiac dilation [76, 77]. Moreover, in cultured cardiomyocytes, overexpression of SOCS3 (limiting IL-6-family signaling) completely suppressed the ability of the IL-6-family cytokines to be anti-apoptotic and to induce hypertrophy. The authors conclude that SOCS3 is a mechanical stress-inducible gene in cardiac muscle cells and that it directly modulates stress-induced gp130 cytokine receptor signaling as the key molecular switch for a negative feedback circuit for both myocyte hypertrophy and survival [78]. Conversely, a decrease in myocardial SOCS3 protein expression, has been found in the left ventricle of patients with dilated cardiomyopathy [79]. IL-6 is also upregulated in the infarcted myocardium and might modulate the inflammatory and reparative response signaling through IL-6 receptor and activating the JAK/STAT cascade [80]. However, the pleiotropic effects of IL-6 in the healing infarct and the induction of other IL-6 family members such as IL-11, oncostatin M and leukemia inhibitory factor that might compensate for the cytokine’s loss, may raise concerns regarding the potential role of IL-6 and its receptor as a therapeutic target in patients with MI remodeling [49]. Despite the well-established pro-inflammatory nature of IL-6 in myocardial ischemia, a recent meta-analysis reports a limited contribution of IL-6 in the cardiac remodeling of hearts in animal models of myocardial ischemia [81]. They showed that genetically deleted IL-6 murine models produced contrasting results. The authors underscored that additional pre-clinical studies exploring the pharmacological inhibition of IL-6R are required to determine the beneficial effects of IL-6 inhibitors in regulating cardiac remodeling and that the findings from IL-6R inhibition have better clinical relevance compared to genetically inhibited IL-6 [81].

The reasons for these apparently discrepant results in relation to the role of IL-6 in myocardial remodeling is at present not clear, but several non-elusive possibilities exist. First, the fine-tuning and timing of the signal cascade of IL-6 that is needed to have beneficial effects on both the infarct phase and the repair process is difficult to mirror in pre-clinical models of knock-out or transgenic animal models. Second, IL-6 is regarded as an inflammatory cytokine and TGF-β as an anti-inflammatory and pro-fibrotic cytokine, seemingly reflecting different effects on the repair process following MI. However, these pathways also interact, and IL-6 has been shown to stimulate myocardial fibrosis through the TGFβ1-Smad-MM2/9 signaling transduction pathway [82], illustrating the complex interaction between the apparent contra dictionary signaling pathways during post-MI remodeling. Intriguingly, Alter C et al. recently showed that post-MI IL-6 was mainly derived from activated cardiac fibroblasts and was controlled by T cell-derived adenosine [83], further underscoring the complex interaction between immune activation and fibrogenesis in infarct healing. In fact, it has been suggested that inhibition of IL-6 production from fibroblast alleviates cardiac remodeling after MI [84], further underscoring the need for targeting therapy. Third, whereas IL-6 levels have been associated with adverse myocardial remodeling following MI [46, 85, 86], firm data on the effect of IL-6 inhibition on post-MI remodeling in patients with coronary artery disease are lacking. In the ASSAIL-MI trial, we have shown that IL-6R inhibition by tocilizumab improved myocardial salvage index (MSI) and microvascular obstruction (MVO) at 3–7 days following MI, of which the latter could be a sign of adverse myocardial remodeling [87]. Importantly, in the ASSAIL-MI trial, the patients received only one dosage of tocilizumab, with the intention to avoid full IL-6 suppression during the infarct healing process. Nonetheless, the effect on MVO was not seen at six months, which weakens the assumption of an effect of transient IL-6R inhibition on myocardial remodeling, underscoring the need for larger clinical studies. Finally, as discussed above, a selective blockade of the inflammatory IL-6 trans-signaling may be more beneficial than a more unselective IL-6/IL-6R blockade [88]. Indeed, by using a novel recombinant fusion-protein sgp130Fc, George et al. showed that selective trans-signaling blocking was more efficacious than pan-antagonism through anti-IL-6 antibodies. In a model of reperfusion after MI in male rats they showed that sgp130Fc reduced infarct size and preserved cardiac function 28-days post-MI, as compared with anti-IL-6 therapy [45].

### IL-6 and Possible Adverse Effects on Lipid Metabolism

IL-6 is known to alter metabolic pathways, and subsequently, IL-6 blockade also alters lipid metabolism [89]. An approximately 10% increase in triglycerides (TG) and LDL cholesterol was observed in patients with rheumatoid arthritis receiving anti-IL-6R treatment by tocilizumab. This was accompanied with reduced inflammation and positive effects on certain lipoprotein-related parameters such as a 50% decrease in lipoprotein(a) (Lp(a)) and a 5% reduction in oxidized LDL levels, with maintained capacity of total HDL and HDL3c to promote cellular cholesterol efflux [89]. Lp(a) as a risk factor for atherosclerosis and related diseases have recently received much attention [90], and the levels of Lp(a) is mediated through expression of the *LPA* gene, which encodes apolipoprotein (a), the unique protein component of Lp(a) [91]. This occurs through IL-6 response elements located in the promoter region of the *LPA* gene. When IL-6 levels are elevated, as seen in chronic inflammation or autoimmune diseases, there is increased activation of these promoter elements, leading to higher synthesis of apolipoprotein(a) and consequently increased Lp(a) concentrations in the blood [92, 93] (Fig. 2). This mechanism may underlie the Lp(a)-lowering effect observed in IL-6 therapies. Notably, however, in contrast to patients with rheumatoid arthritis, Lp(a) was not modified by IL-6R inhibition by tocilizumab in patients with non-ST-elevation MI (NSTEMI) [94]. Further, in contrast to the increasing effect of IL-6R inhibition in patients with rheumatoid arthritis, the effect of tocilizumab on LDL levels in both NSTEMI and STEMI patients treated with tocilizumab were limited [47, 87]. However, a transient increase in TG was also observed in the ASSAIL-MI study [87]. The differences in the metabolic responses to anti-IL6R response between patients with autoimmune diseases and NSTEMI/STEMI patients are not clear, but could include more use of statins in the MI patients that reduce LDL levels, but may increase Lp(a) levels [95]. Also, in contrast to patients with autoimmune disorders receiving repeated dosages of anti-IL-6R therapy, MI patients received one dosage. The clinical significance of these changes for cardiovascular risk is however still under investigation, and some studies suggest the qualitative risk may not increase proportionally to the rise in lipid levels [96]. Notably, in our NSTEMI study, tocilizumab reduced PCSK9 levels in patients with a previous atherogenic lipid profile, further illustrating the complexity of the interplay between IL-6 and lipids [97]. Similar to tocilizumab, ziltivekimab, a monoclonal antibody targeting IL-6, showed minor effects on lipid levels with no effects on total cholesterol to HDL cholesterol ratios and a small, but significant, increase in TG levels in patients with or at a risk for chronic atherosclerotic disease [98]. However, in contrast to the tocilizumab study with NSTEMI patients [94] they reported a decrease in Lp(a) [98]. The reason for this discrepancy is at present not clear but could involve different patient populations (i.e., NSTEMI versus chronic renal impairment) and the duration of treatment (i.e., one dose versus treatment for 12 weeks).

### IL-6: Potential Harmful Effects on Atherothrombosis

Formation of atherothrombosis is a crucial mechanism underlying development of atherosclerotic-related clinical events such as acute coronary syndrome (ACS) and ischemic stroke [99–102]. IL-6 contributes to development of atherothrombosis through multiple mechanisms. The endothelium plays a central role in regulating hemostasis, expressing both pro- and anti-coagulants and pro- and anti-adhesion molecules, and by modulating these molecules, IL-6 can convert the endothelium to a prothrombotic surface [103, 104]. The IL-6-mediated inflammatory response in the endothelium will enhance interaction between platelets and the vascular wall, further promoting atherothrombosis [104, 105]. Through interaction with platelets, IL-6 induce the release of thrombopoietin, enhancing thrombopoiesis in bone marrow resulting in thrombocytosis, which will further pre-dispose to atherothrombosis [106]. It has also been suggested that IL-6, through trans-signaling, will render the platelets more sensitive to other pro-thrombotic stimuli such as thrombin [107] and this priming effect seems to mediated trough enhanced Glycoprotein VI (GPVI)-mediated signaling, a platelet receptor that is activated by collagen [108]. Moreover, IL-6 is an inducer of von Willebrand factor (vWF), tissue factor (TF) and factor VIII, all potent pro-coagulant factors [109, 110], further supporting an enhancing effects of IL-6 as a key mediator of atherothrombosis.

Recently, Ridker et al. conducted a phase 2 study evaluating placebo and versus varying doses of ziltivekimab, directed against the IL-6 ligand, in patients with renal failure and high atherosclerotic risk. The study demonstrated a significant reduction in CRP following ziltivekimab treatment; however no direct data on atherothrombosis were reported [107]. More recently, however, Ministrini et al. showed that ziltivekimab blunted thrombus formation in mice with low-grade chronic inflammation and that platelet reactivity from patients with previous percutaneous coronary intervention (PCI) fell significantly after ex vivo treatment with ziltivekimab [111]. In the ASSAIL-MI trial, tocilizumab induced a modest decrease in platelet count at 3–7 days following hospital admission of STEMI patients compared with placebo, but with no effects on platelet and endothelial-derived CXC-chemokines [112]. So far, data on other pro-coagulant factors in this study are lacking.

## Treatment Targeting IL-6 in CVD

### Effect of IL-6 Inhibition in Chronic Atherosclerosis

During the recent years, several publications have reported beneficial effects of ziltivekimab in patients at risk of or living with chronic atherosclerotic disease. Ridker et al. showed that in patients with elevated hsCRP and chronic kidney disease, ziltivekimab dose-dependently reduced hsCRP and other acute phase proteins (i.e., fibrinogen, serum amyloid A and haptoglobin) as well as secretory phospholipase A2 and Lp (a) as compared with placebo [113]. In a *post hoc* analysis they showed that ziltivekimab reduced neutrophil/lymphocyte ratio, suggesting a broad effect on both innate and adaptive immunity [114]. In a study from Japan, the authors confirmed the findings from Ridker et al. by showing that ziltivekimab reduced hsCRP, serum amyloid A and fibrinogen levels, with no significant changes in total cholesterol to HDL cholesterol ratio and a small increase in TG [98]. So far, we lack data on clinical outcome in the ziltivekimab studies, but studies are ongoing in high-risk patients with a composite primary outcome defined as first major adverse cardiovascular (CV) event ((MACE); defined as nonfatal MI, nonfatal stroke, or CV death) (ClinicalTrials.gov ID NCT05021835).

### Effect of IL-6 Inhibition in Acute Myocardial Infarction

Studies on MI have shown that up to 50% of the myocardial damage is caused by the I/R injury [115]. Even though the treatment of MI with PCI has ameliorated the survival rate majorly, there are no approved treatments to dampen the inflammation and I/R injury in MI [116]. However, during the recent years, tocilizumab given as one dose at hospital admission before/during PCI have shown beneficial effects in NSTEMI [117] and STEMI [87]. In patients with NSTEMI, tocilizumab was shown to significantly reduce hsCRP and TnT as compared with placebo, and in the patients with STEMI, tocilizumab had beneficial effect of myocardial salvage index (MSI) and microvascular obstruction (MVO). However, although the tocilizumab studies had myocardial outcome data, as in the ziltivekimab studies, the tocilizumab studies lacked clinical outcome data and larger phase 3 studies are urgently needed. Moreover, in the ASSAIL-MI trial we used one dose of tocilizumab to avoid damping of potential beneficial effects of inflammation during the repair process. However, we used a fixed rather low dose (280 mg) and forthcoming studies should examine if the effect of higher dosage is more beneficial like those who are used in autoimmune disorders (8 mg/kg).

### Possible Mechanisms of Action of IL-6R Inhibition in MI: Downregulation of Complement Receptor C5aR

Several sub-studies have been performed to clarify the mechanisms of action of IL-6R inhibition in MI. In the NSTEMI study, tocilizumab had no or only minor effects on a range of cytokines in plasma except for an increase in levels of interferon-inducing protein 10 (IP-10)/CXCL10 and macrophage inflammatory protein (MIP) 1β/CCL4, suggesting that the beneficial effects of tocilizumab in these patients is not secondary to modulation of the measured mediators [118]. These data may differ from a recent study by Prapiadou et al. suggesting CXCL10 as a potentially downstream causal mediator for IL-6 signaling in atherosclerosis [119]. This study, however, used an integrating multiomics approach that may not necessarily mimic the situation in vivo in NSTEMI patients receiving IL-6R inhibition. Orrem et al. found that tocilizumab significantly attenuated the expression of the complement receptors C5aR1 and C5aR2 in whole blood from NSTEMI patients, and in a parallel experiment, they found that these receptors and in particular C5aR1 were significantly upregulated in peripheral mononuclear cells from STEMI patients [120]. C5aR1 has in various pre-clinical models been shown to promote harmful effects on the myocardium during ischemia [121], and the interaction between IL-6 and this pro-inflammatory receptor is clearly of interest.

### Possible Mechanisms of Action of IL-6RInhibition in MI: Downregulation of Inflammatory Transcripts in Neutrophils and Up-regulation in Monocytes by Tocilizumab

IL-6 increases the release of neutrophils into the circulation, which can contribute to inflammatory responses in various disorders [122]. In the ASSAIL-MI study, tocilizumab induced a decrease in circulating neutrophils as compared with placebo [87], and notably, transcriptome analyses of neutrophils showed markedly down-regulation of several inflammatory related transcripts in the tocilizumab group, pointing to an inhibitory effects of neutrophil degranulation, potentially mitigating cardiac injury [123]. Indeed, high neutrophil levels 24 h post-PCI correlated with elevated TnT, suggesting a harmful inflammatory response that was attenuated by tocilizumab [87, 123]. Moreover, IL-6R inhibition with tocilizumab showed a reduction of NETosis, further supporting a beneficial effect of this treatment modality on neutrophils in patients with STEMI [124].

In the ASSAIL-MI study there was an increase in monocyte counts during hospitalization that was not seen in the tocilizumab group and notably, lower monocyte levels at 24 h were associated with lower TnT levels and higher MSI [87, 125]. Moreover, in contrast to the transcriptome profile in neutrophils, most of the transcripts were up-regulated in monocytes in the tocilizumab group, potentially representing the induction of a protective monocyte sub-type that did not fit into the classical defined subtypes (i.e., classical, intermediate and non-classical) [125, 126]. Indeed, monocyte gene expression suggested that tocilizumab modulated cytokine signaling pathways related to myocardial remodeling, apoptosis, and chemotaxis. These effects seemed to be mediated through a decrease in SOCS3 [125], the primary feedback inhibitor of IL-6 family signaling, controlling the duration of IL-6 signaling and contribute to the cellular response to IL-6 [39]. Of particular interest, in vitro experiments showed that tocilizumab attenuated apoptosis also in I/R exposed rat cardiomyocytes, suggesting the tocilizumab could have direct beneficial effects in the myocardium [125].

### Possible Mechanisms of Action of IL-6R Inhibition in MI: Modulation of Circular RNA Profile and N-6 Methyladenosine Dynamics

Circular (circ) RNAs are non-coding RNAs with important functions in the nervous system, cardiovascular system, and cancer [127]. Their role in MI is poorly described, but the roles of circRNA in apoptosis, inflammation, oxidative stress and angiogenesis [128–131] may suggest a role for circRNA also in this condition. In a sub-study of the ASSAIL-MI trial we showed that tocilizumab increased certain circRNAs (i.e., circUBAC2 and circANKRD42), correlating to the peak TnT-levels and for circUBAC2, also with the final infarct size [132]. Interestingly, our analyses also suggested a potential relevance for the regulated circRNAs for processes like inflammation and apoptosis [132].

Epitranscriptomics, which involves how the environment modulates RNA, is a fascinating new area of research that open for novel prevention and treatment options [133]. The post-transcriptional RNA modification where methylation of the adenosine base at the nitrogen-6 position, forming N6-methyladenosine (m^6^A) RNA, is the most prevalent of the reversible epitranscriptomic modifications in mammals [134]. In a hypothesis-generating sub-study of the ASSAIL-MI trial we recently showed that compared with healthy controls, whole blood from patients with STEMI had a strikingly different pattern of m^6^A, and this response was, at least to some degree, modulated by blocking the IL-6 receptor, including some of the enzymes that regulate m^6^A dynamics [135]. The data on IL-6 and circRNA and m6A must be regarded as preliminary, but they point to a novel area of research in relation to CVD including the effects of IL-6.

### Sex-differences in IL-6 Response – Implications for Anti-inflammatory Treatment of CVD

There is a great need for increased focus on sex-differences in CVD, and although slowly, the attention is growing. Women and men are physiologically different, resulting in distinct patterns in prevalence, symptomatology, pathogenesis, and response to treatment of CVD [136, 137]. There is an urgent need to identify the molecular mechanism underlying these sex-differences to improve prevention and treatment for both women and men, and to deviate from the long-standing “one-size-fits-all” approach in cardiovascular research and care. There are further clear sex-differences in immune responses [138], and these differences seem also to extend to IL-6 responses [139]. In the ASSAIL-MI trial, post-hoc analysis revealed that neutrophilia was more pronounced in men than women in STEMI, and tocilizumab reduced blood neutrophil counts and infarct size to a greater extent in men than women [140]. A similar pattern has also been observed during anti-IL-6R therapy in COVID-19, with a greater benefit in preventing progression to respiratory failure or death in males [141]. These findings underscore the importance of incorporating sex as a biological variable in forthcomming CVD research including therapeutic trials.

### Neuroprotective Role of Targeting the IL-6-system

In addition to MI, ischemic stroke is an atherothrombotic mediated disease in which inflammation play a major role [142–144]. Multiple lines of evidence underscore the therapeutic potential of targeting the IL-6 system in ischemic stroke. First, population-based prospective cohort studies demonstrate that elevated IL-6 levels correlate with increased stroke risk [62]. Second, higher serum IL-6 levels at hospital admission predict worse clinical outcomes in subacute ischemic stroke patients [145, 146]. Third, IL-6 serves as a biomarker for carotid plaque severity, vulnerability, and progression – critical factors in stroke pathogenesis – with these lesions exhibiting strong IL-6 and receptor expression [147]. Fourth, preclinical studies reveal that IL-6 inhibition attenuates brain atrophy and cellular necrosis in stroke models [148]. Despite the compelling body of evidence, clinical data supporting IL-6-targeted therapy in acute ischemic stroke remain scarce.

Recently, the IRIS trial has shown promising effects on reducing microvascular obstruction and improving perfusion of salvaged brain tissue [149] following a single dose of tocilizumab [150]. Meanwhile, the ILLUMINATE study aims to evaluate different dosing regimens of tocilizumab, with magnetic resonance imaging (MRI) assessments scheduled at 2, 24, and 72 h after endovascular thrombectomy. Additionally, the ILLUMINATE trial will conduct *in-depth* molecular characterization of the inflammatory response both during the acute phase and during the post-thrombectomy period (EU CT number 2025–521269-28-00). In the coming years, we foresee that anti-inflammatory therapies including therapy targeting IL-6/IL-6R axis also will become an integral part of ischemic stroke management.

## Conclusion

Therapeutic strategies targeting the IL-6 system in atherothrombosis including MI and ischemic stroke represent a promising but still evolving therapeutic approach. To enhance the precision of patient treatment, it is crucial to develop a comprehensive understanding of the complexity of IL-6 signaling. Emerging evidence supports the potential of promising agents, such as tocilizumab and ziltivekimab, which modulate IL-6 signaling through distinct mechanisms. In particular, there is growing interest in therapies that selectively inhibit IL-6 trans-signaling, which may offer anti-inflammatory benefits while preserving protective functions mediated by classical signaling. Novel treatment strategies that selectively block IL-6 trans-signaling are also needed. Ongoing clinical trials are poised to address existing gaps in our knowledge of the IL-6 system and larger studies with clinical endpoints are also urgently needed. The results of these studies have the potential to reshape clinical practice in the management of cardiovascular disease.

## Key References

- Qu L, Matz AJ, Karlinsey K, Cao Z, Vella AT, Zhou B. Macrophages at the Crossroad of Meta-Inflammation and Inflammaging. Genes (Basel). 2022;13(11).
  - Comprehensive updated review on the intricate interactions between metabolism and aging through inflammation.
- Rose-John S, Jenkins BJ, Garbers C, Moll JM, Scheller J. Targeting IL-6 trans-signalling: past, present and future prospects. Nat Rev Immunol. 2023;23(10):666-81.
  - A comprehensive review on the different forms of IL-6 signaling.
- George MJ, Jasmin NH, Cummings VT, Richard-Loendt A, Launchbury F, Woollard K, et al. Selective Interleukin-6 Trans-Signaling Blockade Is More Effective Than Panantagonism in Reperfused Myocardial Infarction. JACC Basic Transl Sci. 2021;6(5):431-43.
  - One of the first studies to show that a selective inhibition of IL-6 transsignaling has better effect on the myocardium than inhibition of both trans and classical IL-6 signaling.
- Khan MS, Talha KM, Maqsood MH, Rymer JA, Borlaug BA, Docherty KF, et al. Interleukin-6 and Cardiovascular Events in Healthy Adults: MESA. JACC Adv. 2024;3(8):101063.
  - A large multiethnic study on the prognostic significance of interleukin 6 in healthy individuals in relation to cardiovascular events.
- Bick AG, Pirruccello JP, Griffin GK, Gupta N, Gabriel S, Saleheen D, et al. Genetic Interleukin 6 Signaling Deficiency Attenuates Cardiovascular Risk in Clonal Hematopoiesis. Circulation. 2020;141(2):124-31.
  - An important study on the interaction between interleukin 6 and clonal hematopoiesis of indeterminate potential Clonal Hematopoiesis of
- Niyonzima N, Bakke SS, Gregersen I, Holm S, Sandanger Ø, Orrem HL, et al. Cholesterol crystals use complement to increase NLRP3 signaling pathways in coronary and carotid atherosclerosis. EBioMedicine. 2020;60:102985.
  - First clinical study to mechanistically link NLRP3 inflammasome activation to acute coronary syndrome.
- Hilgendorf I, Frantz S, Frangogiannis NG. Repair of the Infarcted Heart: Cellular Effectors, Molecular Mechanisms and Therapeutic Opportunities. Circ Res. 2024;134(12):1718-51.
  - A comprehensive article on the complex molecular pathways that are activated during the repair process following myocardial infarction with focus on potential novel targets for therapy.
- Broch K, Anstensrud AK, Woxholt S, Sharma K, Tøllefsen IM, Bendz B, et al. Randomized Trial of Interleukin-6 Receptor Inhibition in Patients With Acute ST-Segment Elevation Myocardial Infarction. J Am Coll Cardiol. 2021;77(15):1845-55.
  - The first study to show beneficial effects of interleukin 6 receptor inhibition on myocardial outcomes in patients with ST elevation myocardial infarction.
- Ministrini S, Liberale L, Puspitasari YM, Han J, Kirmes K, Unkelbach LP, et al. Direct Interleukin-6 Inhibition Blunts Arterial Thrombosis by Reducing Collagen-Mediated Platelet Activation. Arterioscler Thromb Vasc Biol. 2025.
  - The first study to show that anti-IL-6 therapy attenuate platelet activation in pre-clinical models and in *ex vivo* testing of platelets from patients that had undergone percutaneous intervention.
- Ridker PM, Devalaraja M, Baeres FMM, Engelmann MDM, Hovingh GK, Ivkovic M, et al. IL-6 inhibition with ziltivekimab in patients at high atherosclerotic risk (RESCUE): a double-blind, randomised, placebo-controlled, phase 2 trial. Lancet. 2021;397(10289):2060-9.
  - The first study to show that interleukin 6 inhibition attenuate inflammation in patients at risk for cardiovascular events.
- Huse C, Anstensrud AK, Michelsen AE, Ueland T, Broch K, Woxholt S, et al. Interleukin-6 inhibition in ST-elevation myocardial infarction: Immune cell profile in the randomised ASSAIL-MI trial. EBioMedicine. 2022;80:104013.
  - The study shows that anti-IL-6 therapy attenuates neutrophil degranulation in STEMI as a potential mechanism for its beneficial effects on the myocardium.
- Kamtchum-Tatuene J, Saba L, Heldner MR, Poorthuis MHF, de Borst GJ, Rundek T, et al. Interleukin-6 Predicts Carotid Plaque Severity, Vulnerability, and Progression. Circ Res. 2022;131(2):e22-e33.
  - A large study convincingly showing that interleukin 6 predicts progression of carotid atherosclerosis.

## Data Availability

No datasets were generated or analysed during the current study.

## References

[1] Li Y, Cao GY, Jing WZ, Liu J, Liu M. Global trends and regional differences in incidence and mortality of cardiovascular disease, 1990–2019: findings from 2019 global burden of disease study. Eur J Prev Cardiol. 2023;30(3):276–86. 36458973 10.1093/eurjpc/zwac285

[2] Martin SS, Aday AW, Allen NB, Almarzooq ZI, Anderson CAM, Arora P, et al. 2025 heart disease and stroke statistics: A report of US and global data from the American heart association. Circulation. 2025;151(8):e41–660. 39866113 10.1161/CIR.0000000000001303 PMC12256702

[3] Mallat Z, Tedgui A. Century of milestones and breakthroughs related to the immune mechanisms of atherosclerosis. Arterioscler Thromb Vasc Biol. 2024;44(5):1002–6. 38657035 10.1161/ATVBAHA.124.319397 PMC11042514

[4] Jonasson L, Holm J, Skalli O, Bondjers G, Hansson GK. Regional accumulations of T cells, macrophages, and smooth muscle cells in the human atherosclerotic plaque. Arteriosclerosis. 1986;6(2):131–8. 2937395 10.1161/01.atv.6.2.131

[5] Hansson GK. Inflammation, atherosclerosis, and coronary artery disease. N Engl J Med. 2005;352(16):1685–95. 15843671 10.1056/NEJMra043430

[6] Ridker PM. Clinician’s guide to reducing inflammation to reduce atherothrombotic risk: JACC review topic of the week. J Am Coll Cardiol. 2018;72(25):3320–31. 30415883 10.1016/j.jacc.2018.06.082

[7] Ridker PM. Residual inflammatory risk: addressing the obverse side of the atherosclerosis prevention coin. Eur Heart J. 2016;37(22):1720–2. 26908943 10.1093/eurheartj/ehw024

[8] Ridker PM, Everett BM, Thuren T, MacFadyen JG, Chang WH, Ballantyne C, et al. Antiinflammatory therapy with Canakinumab for atherosclerotic disease. N Engl J Med. 2017;377(12):1119–31. 28845751 10.1056/NEJMoa1707914

[9] Ridker PM, Libby P, MacFadyen JG, Thuren T, Ballantyne C, Fonseca F, et al. Modulation of the interleukin-6 signalling pathway and incidence rates of atherosclerotic events and all-cause mortality: analyses from the Canakinumab Anti-Inflammatory Thrombosis Outcomes Study (CANTOS). Eur Heart J. 2018;39(38):3499–507. 30165610 10.1093/eurheartj/ehy310

[10] Ridker PM, Rane M. Interleukin-6 signaling and anti-Interleukin-6 therapeutics in cardiovascular disease. Circ Res. 2021;128(11):1728–46. 33998272 10.1161/CIRCRESAHA.121.319077

[11] Grebenciucova E, VanHaerents S. Interleukin 6: at the interface of human health and disease. Front Immunol. 2023;14:1255533. 37841263 10.3389/fimmu.2023.1255533 PMC10569068

[12] Kiecolt-Glaser JK, Preacher KJ, MacCallum RC, Atkinson C, Malarkey WB, Glaser R. Chronic stress and age-related increases in the proinflammatory cytokine IL-6. Proc Natl Acad Sci U S A. 2003;100(15):9090–5. 12840146 10.1073/pnas.1531903100 PMC166443

[13] Yamauchi-Takihara K, Ihara Y, Ogata A, Yoshizaki K, Azuma J, Kishimoto T. Hypoxic stress induces cardiac myocyte-derived interleukin-6. Circulation. 1995;91(5):1520–4. 7867193 10.1161/01.cir.91.5.1520

[14] Ancey C, Corbi P, Froger J, Delwail A, Wijdenes J, Gascan H, et al. Secretion of IL-6, IL-11 and LIF by human cardiomyocytes in primary culture. Cytokine. 2002;18(4):199–205. 12126642 10.1006/cyto.2002.1033

[15] Erta M, Quintana A, Hidalgo J. Interleukin-6, a major cytokine in the central nervous system. Int J Biol Sci. 2012;8(9):1254–66. 23136554 10.7150/ijbs.4679 PMC3491449

[16] Aliyu M, Zohora FT, Anka AU, Ali K, Maleknia S, Saffarioun M, et al. Interleukin-6 cytokine: an overview of the immune regulation, immune dysregulation, and therapeutic approach. Int Immunopharmacol. 2022;111:109130. 35969896 10.1016/j.intimp.2022.109130

[17] Tanaka T, Narazaki M, Kishimoto T. IL-6 in inflammation, immunity, and disease. Cold Spring Harb Perspect Biol. 2014;6(10):a016295. 25190079 10.1101/cshperspect.a016295 PMC4176007

[18] Villar-Fincheira P, Sanhueza-Olivares F, Norambuena-Soto I, Cancino-Arenas N, Hernandez-Vargas F, Troncoso R, et al. Role of Interleukin-6 in vascular health and disease. Front Mol Biosci. 2021;8:641734. 33786327 10.3389/fmolb.2021.641734 PMC8004548

[19] Choy EH, De Benedetti F, Takeuchi T, Hashizume M, John MR, Kishimoto T. Translating IL-6 biology into effective treatments. Nat Rev Rheumatol. 2020;16(6):335–45. 32327746 10.1038/s41584-020-0419-z PMC7178926

[20] Covarrubias AJ, Horng T. IL-6 strikes a balance in metabolic inflammation. Cell Metab. 2014;19(6):898–9. 24896536 10.1016/j.cmet.2014.05.009 PMC4103792

[21] Tylutka A, Walas Ł, Zembron-Lacny A. Level of IL-6, TNF, and IL-1β and age-related diseases: a systematic review and meta-analysis. Front Immunol. 2024;15:1330386. 38495887 10.3389/fimmu.2024.1330386 PMC10943692

[22] Zhao Z, Zhang J, Wu Y, Xie M, Tao S, Lv Q, et al. Plasma IL-6 levels and their association with brain health and dementia risk: A population-based cohort study. Brain Behav Immun. 2024;120:430–8. 38897328 10.1016/j.bbi.2024.06.014

[23] Jia X, Buckley L, Sun C, Al Rifai M, Yu B, Nambi V, et al. Association of interleukin-6 and interleukin-18 with cardiovascular disease in older adults: Atherosclerosis Risk in Communities study. Eur J Prev Cardiol. 2023;30(16):1731–40. 37306504 10.1093/eurjpc/zwad197 PMC10637765

[24] Rea IM, Gibson DS, McGilligan V, McNerlan SE, Alexander HD, Ross OA. Age and Age-Related Diseases: Role of Inflammation Triggers and Cytokines. Front Immunol. 2018;9:586. 29686666 10.3389/fimmu.2018.00586 PMC5900450

[25] Jones BE, Maerz MD, Buckner JH. IL-6: a cytokine at the crossroads of autoimmunity. Curr Opin Immunol. 2018;55:9–14. 30248523 10.1016/j.coi.2018.09.002 PMC6286200

[26] Lippitz BE, Harris RA. Cytokine patterns in cancer patients: a review of the correlation between Interleukin 6 and prognosis. Oncoimmunology. 2016;5(5):e1093722. 27467926 10.1080/2162402X.2015.1093722 PMC4910721

[27] Valletta S, Thomas A, Meng Y, Ren X, Drissen R, Sengül H, et al. Micro-environmental sensing by bone marrow stroma identifies IL-6 and TGFβ1 as regulators of hematopoietic ageing. Nat Commun. 2020;11(1):4075. 32796847 10.1038/s41467-020-17942-7 PMC7427787

[28] Fuster JJ, Walsh K. The good, the bad, and the ugly of interleukin-6 signaling. EMBO J. 2014;33(13):1425–7. 24850773 10.15252/embj.201488856 PMC4194086

[29] Gabay C. Interleukin-6 and chronic inflammation. Arthritis Res Ther. 2006;8(Suppl 2):S3. 16899107 10.1186/ar1917 PMC3226076

[30] Franceschi C, Garagnani P, Parini P, Giuliani C, Santoro A. Inflammaging: a new immune-metabolic viewpoint for age-related diseases. Nat Rev Endocrinol. 2018;14(10):576–90. 30046148 10.1038/s41574-018-0059-4

[31] Qu L, Matz AJ, Karlinsey K, Cao Z, Vella AT, Zhou B. Macrophages at the crossroad of meta-inflammation and inflammaging. Genes. 2022. 10.3390/genes13112074. 36360310 10.3390/genes13112074 PMC9690997

[32] Steensberg A, Fischer CP, Keller C, Møller K, Pedersen BK. IL-6 enhances plasma IL-1ra, IL-10, and cortisol in humans. Am J Physiol Endocrinol Metab. 2003;285(2):E433–7. 12857678 10.1152/ajpendo.00074.2003

[33] Gao B, Ahmad MF, Nagy LE, Tsukamoto H. Inflammatory pathways in alcoholic steatohepatitis. J Hepatol. 2019;70(2):249–59. 30658726 10.1016/j.jhep.2018.10.023 PMC6361545

[34] Wang MJ, Zhang HL, Chen F, Guo XJ, Liu QG, Hou J. The double-edged effects of IL-6 in liver regeneration, aging, inflammation, and diseases. Exp Hematol Oncol. 2024;13(1):62. 38890694 10.1186/s40164-024-00527-1 PMC11184755

[35] Kang S, Tanaka T, Narazaki M, Kishimoto T. Targeting interleukin-6 signaling in clinic. Immunity. 2019;50(4):1007–23. 30995492 10.1016/j.immuni.2019.03.026

[36] Uciechowski P, Dempke WCM. Interleukin-6: a masterplayer in the cytokine network. Oncology. 2020;98(3):131–7. 31958792 10.1159/000505099

[37] Jostock T, Müllberg J, Özbek S, Atreya R, Blinn G, Voltz N, et al. Soluble gp130 is the natural inhibitor of soluble interleukin-6 receptor transsignaling responses. Eur J Biochem. 2001;268(1):160–7. 11121117 10.1046/j.1432-1327.2001.01867.x

[38] Heink S, Yogev N, Garbers C, Herwerth M, Aly L, Gasperi C, et al. Trans-presentation of IL-6 by dendritic cells is required for the priming of pathogenic TH17 cells. Nat Immunol. 2017;18(1):74–85. 27893700 10.1038/ni.3632 PMC5164931

[39] Ramadan MM, Kodama M, Mitsuma W, Ito M, Kashimura T, Ikrar T, et al. Impact of percutaneous coronary intervention on the levels of interleukin-6 and C-reactive protein in the coronary circulation of subjects with coronary artery disease. Am J Cardiol. 2006;98(7):915–7. 16996873 10.1016/j.amjcard.2006.04.034

[40] Reeh H, Rudolph N, Billing U, Christen H, Streif S, Bullinger E, et al. Response to IL-6 trans- and IL-6 classic signalling is determined by the ratio of the IL-6 receptor α to gp130 expression: fusing experimental insights and dynamic modelling. Cell Commun Signal. 2019;17(1):46. 31101051 10.1186/s12964-019-0356-0 PMC6525395

[41] Moresi V, Adamo S, Berghella L. The JAK/STAT pathway in skeletal muscle pathophysiology. Front Physiol. 2019;10:500. 31114509 10.3389/fphys.2019.00500 PMC6502894

[42] Fung KY, Louis C, Metcalfe RD, Kosasih CC, Wicks IP, Griffin MDW, et al. Emerging roles for IL-11 in inflammatory diseases. Cytokine. 2022;149:155750. 34689057 10.1016/j.cyto.2021.155750

[43] Schreiber S, Aden K, Bernardes JP, Conrad C, Tran F, Höper H, et al. Therapeutic Interleukin-6 Trans-signaling Inhibition by Olamkicept (sgp130Fc) in patients with active inflammatory bowel disease. Gastroenterology. 2021;160(7):2354–e6611. 33667488 10.1053/j.gastro.2021.02.062

[44] Rose-John S, Jenkins BJ, Garbers C, Moll JM, Scheller J. Targeting IL-6 trans-signalling: past, present and future prospects. Nat Rev Immunol. 2023;23(10):666–81. 37069261 10.1038/s41577-023-00856-y PMC10108826

[45] George MJ, Jasmin NH, Cummings VT, Richard-Loendt A, Launchbury F, Woollard K, et al. Selective Interleukin-6 trans-signaling blockade is more effective than panantagonism in reperfused myocardial infarction. JACC: Basic to Translational Science. 2021;6(5):431–43. 34095633 10.1016/j.jacbts.2021.01.013 PMC8165121

[46] Mehta NN, deGoma E, Shapiro MD. IL-6 and cardiovascular risk: a narrative review. Curr Atheroscler Rep. 2024;27(1):12. 39589436 10.1007/s11883-024-01259-7 PMC11599326

[47] Katkenov N, Mukhatayev Z, Kozhakhmetov S, Sailybayeva A, Bekbossynova M, Kushugulova A. Systematic review on the role of IL-6 and IL-1β in cardiovascular diseases. J Cardiovasc Dev Dis. 2024. 10.3390/jcdd11070206. 39057626 10.3390/jcdd11070206 PMC11277031

[48] Yang C, Deng Z, Li J, Ren Z, Liu F. Meta-analysis of the relationship between interleukin-6 levels and the prognosis and severity of acute coronary syndrome. Clinics (Sao Paulo). 2021;76:e2690. 34231707 10.6061/clinics/2021/e2690 PMC8240769

[49] Feng Y, Ye D, Wang Z, Pan H, Lu X, Wang M, et al. The role of Interleukin-6 family members in cardiovascular diseases. Front Cardiovasc Med. 2022;9:818890. 35402550 10.3389/fcvm.2022.818890 PMC8983865

[50] Khan MS, Talha KM, Maqsood MH, Rymer JA, Borlaug BA, Docherty KF, et al. Interleukin-6 and cardiovascular events in healthy adults: MESA. JACC Adv. 2024;3(8):101063. 39077632 10.1016/j.jacadv.2024.101063 PMC11284704

[51] Ketelhuth DFJ, Bäck M. Myeloid-specific interleukin-6 response: from vascular effects to the potential for novel personalized medicines. Eur Heart J Open. 2024;4(4):oeae047. 39015380 10.1093/ehjopen/oeae047 PMC11250221

[52] Wang L, Ozark PA, Smith ER, Zhao Z, Marshall SA, Rendleman EJ, et al. TET2 coactivates gene expression through demethylation of enhancers. Sci Adv. 2018;4(11):eaau6986. 30417100 10.1126/sciadv.aau6986 PMC6221537

[53] Pasupuleti SK, Ramdas B, Burns SS, Palam LR, Kanumuri R, Kumar R, et al. Obesity-induced inflammation exacerbates clonal hematopoiesis. J Clin Invest. 2023. 10.1172/JCI163968. 37071471 10.1172/JCI163968 PMC10231999

[54] Jaiswal S, Fontanillas P, Flannick J, Manning A, Grauman PV, Mar BG, et al. Age-related clonal hematopoiesis associated with adverse outcomes. N Engl J Med. 2014;371(26):2488–98. 25426837 10.1056/NEJMoa1408617 PMC4306669

[55] Bick AG, Pirruccello JP, Griffin GK, Gupta N, Gabriel S, Saleheen D, et al. Genetic interleukin 6 signaling deficiency attenuates cardiovascular risk in clonal hematopoiesis. Circulation. 2020;141(2):124–31. 31707836 10.1161/CIRCULATIONAHA.119.044362 PMC7008855

[56] Woo J, Lu D, Lewandowski A, Xu H, Serrano P, Healey M, et al. Effects of IL-1β Inhibition on anemia and clonal hematopoiesis in the randomized CANTOS trial. Blood Adv. 2023;7(24):7471–84. 37934948 10.1182/bloodadvances.2023011578 PMC10758744

[57] Jaiswal S, Natarajan P, Silver AJ, Gibson CJ, Bick AG, Shvartz E, et al. Clonal hematopoiesis and risk of atherosclerotic cardiovascular disease. N Engl J Med. 2017;377(2):111–21. 28636844 10.1056/NEJMoa1701719 PMC6717509

[58] Fuster JJ, MacLauchlan S, Zuriaga MA, Polackal MN, Ostriker AC, Chakraborty R, et al. Clonal hematopoiesis associated with TET2 deficiency accelerates atherosclerosis development in mice. Science. 2017;355(6327):842–7. 28104796 10.1126/science.aag1381 PMC5542057

[59] Nordeng J, Schandiz H, Solheim S, Åkra S, Hoffman P, Roald B, et al. The inflammasome signaling pathway is actively regulated and related to myocardial damage in coronary thrombi from patients with STEMI. Mediators Inflamm. 2021;2021:5525917. 34135690 10.1155/2021/5525917 PMC8178014

[60] Lafuse WP, Wozniak DJ, Rajaram MVS. Role of cardiac macrophages on cardiac inflammation, fibrosis and tissue repair. Cells. 2020. 10.3390/cells10010051. 33396359 10.3390/cells10010051 PMC7824389

[61] Libby P, Ridker PM. Inflammation and atherosclerosis: role of C-reactive protein in risk assessment. Am J Med. 2004;116(Suppl 6A):s9–16. 10.1016/j.amjmed.2004.02.006 15050187

[62] Papadopoulos A, Palaiopanos K, Björkbacka H, Peters A, de Lemos JA, Seshadri S, et al. Circulating interleukin-6 levels and incident ischemic stroke: a systematic review and meta-analysis of prospective studies. Neurology. 2022;98(10):e1002-12. 34969940 10.1212/WNL.0000000000013274 PMC8967391

[63] Yang Y, Wang H, Kouadir M, Song H, Shi F. Recent advances in the mechanisms of NLRP3 inflammasome activation and its inhibitors. Cell Death Dis. 2019;10(2):128. 30755589 10.1038/s41419-019-1413-8 PMC6372664

[64] Olsen MB, Gregersen I, Sandanger Ø, Yang K, Sokolova M, Halvorsen BE, et al. Targeting the inflammasome in cardiovascular disease. JACC Basic Transl Sci. 2022;7(1):84–98. 35128212 10.1016/j.jacbts.2021.08.006 PMC8807732

[65] Niyonzima N, Bakke SS, Gregersen I, Holm S, Sandanger Ø, Orrem HL, et al. Cholesterol crystals use complement to increase NLRP3 signaling pathways in coronary and carotid atherosclerosis. EBioMedicine. 2020;60:102985. 32927275 10.1016/j.ebiom.2020.102985 PMC7494683

[66] Cahill CM, Rogers JT. Interleukin (IL) 1beta induction of IL-6 is mediated by a novel phosphatidylinositol 3-kinase-dependent AKT/IkappaB kinase alpha pathway targeting activator protein-1. J Biol Chem. 2008;283(38):25900–12. 18515365 10.1074/jbc.M707692200 PMC2533786

[67] Tosato G, Jones KD. Interleukin-1 induces interleukin-6 production in peripheral blood monocytes. Blood. 1990;75(6):1305–10. 2310829

[68] Li T, Yan Z, Fan Y, Fan X, Li A, Qi Z, et al. Cardiac repair after myocardial infarction: A two-sided role of inflammation-mediated. Front Cardiovasc Med. 2022;9:1077290. 36698953 10.3389/fcvm.2022.1077290 PMC9868426

[69] Matter MA, Paneni F, Libby P, Frantz S, Stähli BE, Templin C, et al. Inflammation in acute myocardial infarction: the good, the bad and the ugly. Eur Heart J. 2024;45(2):89–103. 37587550 10.1093/eurheartj/ehad486 PMC10771378

[70] Zuurbier CJ, Abbate A, Cabrera-Fuentes HA, Cohen MV, Collino M, De Kleijn DPV, et al. Innate immunity as a target for acute cardioprotection. Cardiovasc Res. 2019;115(7):1131–42. 30576455 10.1093/cvr/cvy304 PMC6529915

[71] Frangogiannis NG, Smith CW, Entman ML. The inflammatory response in myocardial infarction. Cardiovasc Res. 2002;53(1):31–47. 11744011 10.1016/s0008-6363(01)00434-5

[72] Hilgendorf I, Frantz S, Frangogiannis NG. Repair of the infarcted heart: cellular effectors, molecular mechanisms and therapeutic opportunities. Circ Res. 2024;134(12):1718–51. 38843294 10.1161/CIRCRESAHA.124.323658 PMC11164543

[73] Frangogiannis NG. Regulation of the inflammatory response in cardiac repair. Circ Res. 2012;110(1):159–73. 22223212 10.1161/CIRCRESAHA.111.243162 PMC3690135

[74] Hanna A, Frangogiannis NG. The role of the TGF-β superfamily in myocardial infarction. Front Cardiovasc Med. 2019;6:140. 31620450 10.3389/fcvm.2019.00140 PMC6760019

[75] Murtha LA, Schuliga MJ, Mabotuwana NS, Hardy SA, Waters DW, Burgess JK, et al. The processes and mechanisms of cardiac and pulmonary fibrosis. Front Physiol. 2017;8:777. 29075197 10.3389/fphys.2017.00777 PMC5643461

[76] Banerjee I, Fuseler JW, Intwala AR, Baudino TA. IL-6 loss causes ventricular dysfunction, fibrosis, reduced capillary density, and dramatically alters the cell populations of the developing and adult heart. Am J Physiol Heart Circ Physiol. 2009;296(5):H1694–704. 19234091 10.1152/ajpheart.00908.2008 PMC2685341

[77] Meléndez GC, McLarty JL, Levick SP, Du Y, Janicki JS, Brower GL. Interleukin 6 mediates myocardial fibrosis, concentric hypertrophy, and diastolic dysfunction in rats. Hypertension. 2010;56(2):225–31. 20606113 10.1161/HYPERTENSIONAHA.109.148635 PMC2921860

[78] Yasukawa H, Hoshijima M, Gu Y, Nakamura T, Pradervand S, Hanada T, et al. Suppressor of cytokine signaling-3 is a biomechanical stress-inducible gene that suppresses gp130-mediated cardiac myocyte hypertrophy and survival pathways. J Clin Invest. 2001;108(10):1459–67. 11714737 10.1172/JCI13939 PMC209425

[79] Podewski EK, Hilfiker-Kleiner D, Hilfiker A, Morawietz H, Lichtenberg A, Wollert KC, et al. Alterations in Janus kinase (JAK)-signal transducers and activators of transcription (STAT) signaling in patients with end-stage dilated cardiomyopathy. Circulation. 2003;107(6):798–802. 12591746 10.1161/01.cir.0000057545.82749.ff

[80] Boengler K, Hilfiker-Kleiner D, Drexler H, Heusch G, Schulz R. The myocardial JAK/STAT pathway: from protection to failure. Pharmacol Ther. 2008;120(2):172–85. 18786563 10.1016/j.pharmthera.2008.08.002

[81] Duddu S, Agrawal M, Chakrabarti R, Ghosh A, Chakravorty N, Tiwari A, et al. Meta-analysis reveals inhibition of the inflammatory cytokine IL-6 affords limited protection post-myocardial ischemia/infarction. Heliyon. 2022;8(8):e10435. 36090222 10.1016/j.heliyon.2022.e10435 PMC9449900

[82] Wang J, Wang M, Lu X, Zhang Y, Zeng S, Pan X, et al. IL-6 inhibitors effectively reverse post-infarction cardiac injury and ischemic myocardial remodeling via the TGF-β1/Smad3 signaling pathway. Exp Ther Med. 2022;24(3):576. 35949328 10.3892/etm.2022.11513 PMC9353402

[83] Alter C, Henseler AS, Owenier C, Hesse J, Ding Z, Lautwein T, et al. IL-6 in the infarcted heart is preferentially formed by fibroblasts and modulated by purinergic signaling. J Clin Invest. 2023. 10.1172/JCI163799. 36943408 10.1172/JCI163799 PMC10232006

[84] Zhang M, Zhang M, Zhou T, Liu M, Xia N, Gu M, et al. Inhibition of fibroblast IL-6 production by ACKR4 deletion alleviates cardiac remodeling after myocardial infarction. Biochem Biophys Res Commun. 2021;547:139–47. 33610913 10.1016/j.bbrc.2021.02.013

[85] Tøllefsen IM, Shetelig C, Seljeflot I, Eritsland J, Hoffmann P, Andersen G. High levels of interleukin-6 are associated with final infarct size and adverse clinical events in patients with STEMI. Open Heart. 2021. 10.1136/openhrt-2021-001869. 34933964 10.1136/openhrt-2021-001869 PMC8693166

[86] Tiller C, Reindl M, Holzknecht M, Lechner I, Schwaiger J, Brenner C, et al. Association of plasma interleukin-6 with infarct size, reperfusion injury, and adverse remodelling after ST-elevation myocardial infarction. Eur Heart J Acute Cardiovasc Care. 2022;11(2):113–23. 34849677 10.1093/ehjacc/zuab110

[87] Broch K, Anstensrud AK, Woxholt S, Sharma K, Tøllefsen IM, Bendz B, et al. Randomized trial of Interleukin-6 receptor Inhibition in patients with acute ST-Segment elevation myocardial infarction. J Am Coll Cardiol. 2021;77(15):1845–55. 33858620 10.1016/j.jacc.2021.02.049

[88] Aukrust P, Kleveland O, Gullestad L. Targeting IL-6 trans-signaling: amplifying the benefits of IL-6 inhibition in myocardial infarction. JACC Basic Transl Sci. 2021;6(5):444–6. 34102669 10.1016/j.jacbts.2021.01.007 PMC8165105

[89] Pierini FS, Botta E, Soriano ER, Martin M, Boero L, Meroño T, et al. Effect of Tocilizumab on LDL and HDL characteristics in patients with rheumatoid Arthritis. An observational study. Rheumatol Ther. 2021;8(2):803–15. 33811316 10.1007/s40744-021-00304-0 PMC8217399

[90] Barkas F, Brandts J, De Bacquer D, Jennings C, De Backer GG, Kotseva K, et al. Global variation in Lipoprotein(a) levels among patients with coronary heart disease: insights from the INTERASPIRE study and implications for emerging Lp(a)-Lowering therapies. J Am Coll Cardiol. 2025;85(21):2028–42. 40436467 10.1016/j.jacc.2025.04.010

[91] Reyes-Soffer G, Yeang C, Michos ED, Boatwright W, Ballantyne CM. High lipoprotein(a): actionable strategies for risk assessment and mitigation. Am J Prev Cardiol. 2024;18:100651. 38646021 10.1016/j.ajpc.2024.100651 PMC11031736

[92] Müller N, Schulte DM, Türk K, Freitag-Wolf S, Hampe J, Zeuner R, et al. IL-6 Blockade by monoclonal antibodies inhibits Apolipoprotein (a) expression and lipoprotein (a) synthesis in humans. J Lipid Res. 2015;56(5):1034–42. 25713100 10.1194/jlr.P052209 PMC4409280

[93] Makris A, Barkas F, Sfikakis PP, Liberopoulos E, Filippatos TD, Ray KK, et al. Lipoprotein(a), Interleukin-6 inhibitors, and atherosclerotic cardiovascular disease: is there an association? Atheroscler Plus. 2023;54:1–6. 37720252 10.1016/j.athplu.2023.09.001 PMC10500445

[94] Ueland T, Kleveland O, Michelsen AE, Wiseth R, Damås JK, Holven KB, et al. Serum lipoprotein(a) is not modified by interleukin-6 receptor antagonism or associated with inflammation in non-ST-elevation myocardial infarction. Int J Cardiol. 2019;274:348–50. 29961573 10.1016/j.ijcard.2018.06.093

[95] Yahya R, Berk K, Verhoeven A, Bos S, van der Zee L, Touw J, et al. Statin treatment increases lipoprotein(a) levels in subjects with low molecular weight apolipoprotein(a) phenotype. Atherosclerosis. 2019;289:201–5. 31327478 10.1016/j.atherosclerosis.2019.07.001

[96] You D, Tang Y, Lange T, Wu Y, Lu M, Shao F, et al. Systematic analysis of relationships between serum lipids with all-cause and cause-specific mortality: evidence from prospective cohort studies of UK biobank and women’s health initiative. Clin Nutr. 2025;47:94–102. 39999642 10.1016/j.clnu.2025.02.009

[97] Ueland T, Kleveland O, Michelsen AE, Wiseth R, Damås JK, Aukrust P, et al. Serum PCSK9 is modified by interleukin-6 receptor antagonism in patients with hypercholesterolaemia following non-ST-elevation myocardial infarction. Open Heart. 2018;5(2):e000765. 30258647 10.1136/openhrt-2017-000765 PMC6150185

[98] Wada Y, Jensen C, Meyer ASP, Zonoozi AAM, Honda H. Efficacy and safety of interleukin-6 inhibition with Ziltivekimab in patients at high risk of atherosclerotic events in Japan (RESCUE-2): a randomized, double-blind, placebo-controlled, phase 2 trial. J Cardiol. 2023;82(4):279–85. 37211246 10.1016/j.jjcc.2023.05.006

[99] Olie RH, van der Meijden PEJ, Ten Cate H. The coagulation system in atherothrombosis: implications for new therapeutic strategies. Res Pract Thromb Haemost. 2018;2(2):188–98. 30046721 10.1002/rth2.12080 PMC6055505

[100] Nardin M, Verdoia M, Laera N, Cao D, De Luca G. New insights into pathophysiology and new risk factors for ACS. J Clin Med. 2023. 10.3390/jcm12082883. 37109221 10.3390/jcm12082883 PMC10146393

[101] Fuster V, Moreno PR, Fayad ZA, Corti R, Badimon JJ. Atherothrombosis and high-risk plaque: part I: evolving concepts. J Am Coll Cardiol. 2005;46(6):937–54. 16168274 10.1016/j.jacc.2005.03.074

[102] Rothwell PM. Atherothrombosis and ischaemic stroke. BMJ. 2007;334(7590):379–80. 17322217 10.1136/bmj.38964.489051.80 PMC1804195

[103] Gross PL, Aird WC. The endothelium and thrombosis. Semin Thromb Hemost. 2000;26(5):463–78. 11129402 10.1055/s-2000-13202

[104] Hou T, Tieu BC, Ray S, Recinos Iii A, Cui R, Tilton RG, et al. Roles of IL-6-gp130 signaling in vascular inflammation. Curr Cardiol Rev. 2008;4(3):179–92. 19936194 10.2174/157340308785160570 PMC2780819

[105] Gawaz M, Langer H, May AE. Platelets in inflammation and atherogenesis. J Clin Invest. 2005;115(12):3378–84. 16322783 10.1172/JCI27196 PMC1297269

[106] Kaser A, Brandacher G, Steurer W, Kaser S, Offner FA, Zoller H, et al. Interleukin-6 stimulates thrombopoiesis through thrombopoietin: role in inflammatory thrombocytosis. Blood. 2001;98(9):2720–5. 11675343 10.1182/blood.v98.9.2720

[107] Helgason H, Sulem P, Duvvari MR, Luo H, Thorleifsson G, Stefansson H, et al. A rare nonsynonymous sequence variant in C3 is associated with high risk of age-related macular degeneration. Nat Genet. 2013;45(11):1371–4. 24036950 10.1038/ng.2740

[108] Webb CE, Vautrinot J, Hers I. IL-6 as a mediator of platelet hyper-responsiveness. Cells. 2025;14(11):766. 40497942 10.3390/cells14110766 PMC12153796

[109] Chin BS, Conway DS, Chung NA, Blann AD, Gibbs CR, Lip GY. Interleukin-6, tissue factor and von Willebrand factor in acute decompensated heart failure: relationship to treatment and prognosis. Blood Coagul Fibrinolysis. 2003;14(6):515–21. 12960603 10.1097/00001721-200309000-00001

[110] Stirling D, Hannant WA, Ludlam CA. Transcriptional activation of the factor VIII gene in liver cell lines by interleukin-6. Thromb Haemost. 1998;79(1):74–8. 9459327

[111] Ministrini S, Liberale L, Puspitasari YM, Han J, Kirmes K, Unkelbach LP, et al. Direct interleukin-6 inhibition blunts arterial thrombosis by reducing collagen-mediated platelet activation. Arterioscler Thromb Vasc Biol. 2025. 10.1161/ATVBAHA.125.322533. 40534557 10.1161/ATVBAHA.125.322533 PMC12278750

[112] Huang L, Zhang Q, Huang X, Qu C, Ma S, Mao Y, et al. Mutation screening in genes known to be responsible for retinitis pigmentosa in 98 small Han Chinese families. Sci Rep. 2017;7(1):1948. 28512305 10.1038/s41598-017-00963-6 PMC5434011

[113] Ridker PM, Devalaraja M, Baeres FMM, Engelmann MDM, Hovingh GK, Ivkovic M, et al. IL-6 Inhibition with Ziltivekimab in patients at high atherosclerotic risk (RESCUE): a double-blind, randomised, placebo-controlled, phase 2 trial. Lancet. 2021;397(10289):2060–9. 34015342 10.1016/S0140-6736(21)00520-1

[114] Adamstein NH, Cornel JH, Davidson M, Libby P, de Remigis A, Jensen C, et al. Association of Interleukin 6 Inhibition with Ziltivekimab and the Neutrophil-Lymphocyte ratio: A secondary analysis of the RESCUE clinical trial. JAMA Cardiol. 2023;8(2):177–81. 36449307 10.1001/jamacardio.2022.4277 PMC9713672

[115] Black L, Coombs VJ, Townsend SN. Reperfusion and reperfusion injury in acute myocardial infarction. Heart Lung. 1990;19(3):274–84. 2187833

[116] Parikh PB, Bhatt DL, Bhasin V, Anker SD, Skopicki HA, Claessen BE, et al. Impact of percutaneous coronary intervention on outcomes in patients with heart failure: JACC State-of-the-Art review. J Am Coll Cardiol. 2021;77(19):2432–47. 33985688 10.1016/j.jacc.2021.03.310

[117] Kleveland O, Kunszt G, Bratlie M, Ueland T, Broch K, Holte E, et al. Effect of a single dose of the interleukin-6 receptor antagonist Tocilizumab on inflammation and troponin T release in patients with non-ST-elevation myocardial infarction: a double-blind, randomized, placebo-controlled phase 2 trial. Eur Heart J. 2016;37(30):2406–13. 27161611 10.1093/eurheartj/ehw171

[118] Kleveland O, Ueland T, Kunszt G, Bratlie M, Yndestad A, Broch K, et al. Interleukin-6 receptor inhibition with tocilizumab induces a selective and substantial increase in plasma IP-10 and MIP-1β in non-ST-elevation myocardial infarction. Int J Cardiol. 2018;271:1–7. 29961572 10.1016/j.ijcard.2018.04.136

[119] Prapiadou S, Živković L, Thorand B, George MJ, van der Laan SW, Malik R, et al. Proteogenomic data integration reveals CXCL10 as a potentially downstream causal mediator for IL-6 signaling on atherosclerosis. Circulation. 2024;149(9):669–83. 38152968 10.1161/CIRCULATIONAHA.123.064974 PMC10922752

[120] Orrem HL, Nilsson PH, Pischke SE, Kleveland O, Yndestad A, Ekholt K, et al. IL-6 receptor inhibition by tocilizumab attenuated expression of C5a receptor 1 and 2 in non-ST-elevation myocardial infarction. Front Immunol. 2018;9:2035. 30258440 10.3389/fimmu.2018.02035 PMC6143659

[121] van der Pals J, Koul S, Andersson P, Götberg M, Ubachs JF, Kanski M, et al. Treatment with the C5a receptor antagonist ADC-1004 reduces myocardial infarction in a porcine ischemia-reperfusion model. BMC Cardiovasc Disord. 2010;10:45. 20875134 10.1186/1471-2261-10-45 PMC2955599

[122] Hashizume M, Higuchi Y, Uchiyama Y, Mihara M. IL-6 plays an essential role in neutrophilia under inflammation. Cytokine. 2011;54(1):92–9. 21292497 10.1016/j.cyto.2011.01.007

[123] Huse C, Anstensrud AK, Michelsen AE, Ueland T, Broch K, Woxholt S, et al. Interleukin-6 Inhibition in ST-elevation myocardial infarction: immune cell profile in the randomised ASSAIL-MI trial. EBioMedicine. 2022;80:104013. 35504178 10.1016/j.ebiom.2022.104013 PMC9079006

[124] Kindberg KM, Broch K, Andersen G, Anstensrud AK, Åkra S, Woxholt S, et al. Neutrophil extracellular traps in ST-Segment elevation myocardial infarction: reduced by Tocilizumab and associated with infarct size. JACC Adv. 2024;3(9):101193. 39247678 10.1016/j.jacadv.2024.101193 PMC11378880

[125] Huse C, Murphy S, Yang K, Balzer N, Stokke M, Anstensrud AK et al. The effects of interleukin-6-receptor Inhibition on monocytes in STEMI: a substudy of the ASSAIL-MI trial. eBiomedicine. 2025; 121;10596. 10.1016/j.ebiom.2025.105960 PMC12547449 41076990

[126] Kapellos TS, Bonaguro L, Gemünd I, Reusch N, Saglam A, Hinkley ER, et al. Human monocyte subsets and phenotypes in major chronic inflammatory diseases. Front Immunol. 2019;10:2035. 31543877 10.3389/fimmu.2019.02035 PMC6728754

[127] He AT, Liu J, Li F, Yang BB. Targeting circular RNAs as a therapeutic approach: current strategies and challenges. Signal Transduct Target Ther. 2021;6(1):185. 34016945 10.1038/s41392-021-00569-5 PMC8137869

[128] Chen W, Zhang Y, Yin M, Cheng Z, Li D, Luo X, et al. Circular RNA circPRDX3 mediates neuronal survival apoptosis in ischemic stroke by targeting miR-641 and NPR3. Brain Res. 2022;1797:148114. 36208650 10.1016/j.brainres.2022.148114

[129] Huang R, Zhang W, Li W, Gao Y, Zheng D, Bi G. Overexpressing circ_0000831 is sufficient to inhibit neuroinflammation and vertigo in cerebral ischemia through a miR-16-5p-dependent mechanism. Exp Neurol. 2022;353:114047. 35300972 10.1016/j.expneurol.2022.114047

[130] Cheng Q, Wang J, Li M, Fang J, Ding H, Meng J, et al. CircSV2b participates in oxidative stress regulation through miR-5107-5p-Foxk1-Akt1 axis in parkinson’s disease. Redox Biol. 2022;56:102430. 35973363 10.1016/j.redox.2022.102430 PMC9396399

[131] Xie Q, Ma Y, Ren Z, Gu T, Jiang Z. Circular RNA: a new expectation for cardiovascular diseases. J Cell Biochem. 2024;125(2):e30512. 38098251 10.1002/jcb.30512

[132] Holme FA, Huse C, Kong XY, Broch K, Gullestad L, Anstensrud AK, et al. Circular RNA profile in atherosclerotic disease: regulation during ST-elevated myocardial infarction. Int J Mol Sci. 2024. 10.3390/ijms25169014. 39201700 10.3390/ijms25169014 PMC11354517

[133] Cerneckis J, Ming GL, Song H, He C, Shi Y. The rise of epitranscriptomics: recent developments and future directions. Trends Pharmacol Sci. 2024;45(1):24–38. 38103979 10.1016/j.tips.2023.11.002 PMC10843569

[134] Yang Y, Hsu PJ, Chen YS, Yang YG. Dynamic transcriptomic m(6)A decoration: writers, erasers, readers and functions in RNA metabolism. Cell Res. 2018;28(6):616–24. 29789545 10.1038/s41422-018-0040-8 PMC5993786

[135] Dahl TB, Quiles-Jiménez A, Broch K, Anstensrud AK, Gullestad L, Andersen G, et al. The N-6 Methyladenosine dynamics in STEMI and the effect of IL-6 inhibition - a hypothesis generating sub-study of the ASSAIL-MI trial. Front Immunol. 2025;16:1532325. 40547027 10.3389/fimmu.2025.1532325 PMC12178860

[136] Rajendran A, Minhas AS, Kazzi B, Varma B, Choi E, Thakkar A, et al. Sex-specific differences in cardiovascular risk factors and implications for cardiovascular disease prevention in women. Atherosclerosis. 2023;384:117269. 37752027 10.1016/j.atherosclerosis.2023.117269 PMC10841060

[137] Vervoort D, Wang R, Li G, Filbey L, Maduka O, Brewer LC, et al. Addressing the global burden of cardiovascular disease in women: JACC State-of-the-Art review. J Am Coll Cardiol. 2024;83(25):2690–707. 38897679 10.1016/j.jacc.2024.04.028

[138] Forsyth KS, Jiwrajka N, Lovell CD, Toothacre NE, Anguera MC. The connexion between sex and immune responses. Nat Rev Immunol. 2024;24(7):487–502. 38383754 10.1038/s41577-024-00996-9 PMC11216897

[139] Mun CJ, Letzen JE, Nance S, Smith MT, Khanuja HS, Sterling RS, et al. Sex Differences in Interleukin-6 Responses Over Time Following Laboratory Pain Testing Among Patients With Knee Osteoarthritis. J Pain. 2020;21(5–6):731–41. 31733364 10.1016/j.jpain.2019.11.003 PMC7217718

[140] Svedlund Eriksson E, Lantero Rodriguez M, Halvorsen B, Johansson I, Mårtensson AKF, Wilhelmson AS, et al. Testosterone exacerbates neutrophilia and cardiac injury in myocardial infarction via actions in bone marrow. Nat Commun. 2025;16(1):1142. 39910039 10.1038/s41467-025-56217-x PMC11799197

[141] Stein DF, Foley C, Byott M, Nastouli E, Ambler G, Arulkumaran N. Biological sex is associated with heterogeneous responses to IL-6 receptor inhibitor treatment in COVID-19-a retrospective cohort study. Sci Rep. 2023;13(1):13504. 37598275 10.1038/s41598-023-40744-y PMC10439929

[142] Shekhar S, Cunningham MW, Pabbidi MR, Wang S, Booz GW, Fan F. Targeting vascular inflammation in ischemic stroke: recent developments on novel immunomodulatory approaches. Eur J Pharmacol. 2018;833:531–44. 29935175 10.1016/j.ejphar.2018.06.028 PMC6090562

[143] Lindsberg PJ, Grau AJ. Inflammation and infections as risk factors for ischemic stroke. Stroke. 2003;34(10):2518–32. 14500942 10.1161/01.STR.0000089015.51603.CC

[144] Michelsen AE, Rathcke CN, Skjelland M, Holm S, Ranheim T, Krohg-Sørensen K, et al. Increased YKL-40 expression in patients with carotid atherosclerosis. Atherosclerosis. 2010;211(2):589–95. 20347092 10.1016/j.atherosclerosis.2010.02.035

[145] Băcilă CI, Vlădoiu MG, Văleanu M, Moga DF, Pumnea PM. The role of IL-6 and TNF-Alpha biomarkers in predicting disability outcomes in acute ischemic stroke patients. Life. 2025. 10.3390/life15010047. 39859987 10.3390/life15010047 PMC11766476

[146] Pawluk H, Grześk G, Kołodziejska R, Kozakiewicz M, Woźniak A, Grzechowiak E, et al. Effect of IL-6 and hsCRP serum levels on functional prognosis in stroke patients undergoing IV-thrombolysis: retrospective analysis. Clin Interv Aging. 2020;15:1295–303. 32821090 10.2147/CIA.S258381 PMC7418453

[147] Kamtchum-Tatuene J, Saba L, Heldner MR, Poorthuis MHF, de Borst GJ, Rundek T, et al. Interleukin-6 predicts carotid plaque severity, vulnerability, and progression. Circ Res. 2022;131(2):e22–33. 35713008 10.1161/CIRCRESAHA.122.320877 PMC9308732

[148] Hall C, Nguyen DT, Mendoza K, Tan C, Chauhan A. Inhibition of IL-6 trans-signaling promotes post-stroke functional recovery in a sex and dose-dependent manner. J Neuroinflammation. 2025;22(1):52. 40011978 10.1186/s12974-025-03365-y PMC11866694

[149] Chu X, Ma Z, Liu Y, Sun J, Wang N, Li C, et al. IRIS, a randomised, double-blind, placebo-controlled trial of interleukin-6 receptor inhibition undergoing endovascular treatment in acute anterior circulation ischaemic stroke: study rationale and design. Stroke Vasc Neurol. 2024. 10.1136/svn-2024-003574. 10.1136/svn-2024-003574 PMC12415643 39608800

[150] Eren F, Yilmaz SE. Neuroprotective approach in acute ischemic stroke: a systematic review of clinical and experimental studies. Brain Circ. 2022;8(4):172–9. 37181847 10.4103/bc.bc_52_22 PMC10167855

